# Supplementary material for: Mapping knowledge gaps in marine diversity reveals a latitudinal gradient of missing species richness
Source: Nat Commun. 2018 Nov 9;9:4713. doi: 10.1038/s41467-018-07217-7 (PMC6226500; doi:10.1038/s41467-018-07217-7)
Supplement: Supplementary file 1 — Supplementary Information [file 41467_2018_7217_MOESM1_ESM.pdf]

Supplementary Information

Menegotto et al.

Mapping knowledge gaps in marine diversity reveals a latitudinal gradient of missing species richness

CONTENTS

Supplementary Note 1 | Latitudinal range of marine species 4

Supplementary Note 2 | Bathymetric distribution of marine species records 5

Supplementary Note 3 | Standardizing sample size and completeness 5

Supplementary Note 4 | OBIS representativeness and inventory completeness estimate 7

Supplementary Note 5 | Data cleaning procedures 9

Supplementary Figure 1 | Latitudinal pattern in species richness according to different data sources 11

Supplementary Figure 2 | Latitudinal pattern in species richness of different taxonomic groups........ 12

Supplementary Figure 3 | Latitudinal pattern in sampling events of each taxonomic group 13

Supplementary Figure 4 | Inventory completeness of each taxonomic group estimated by the sample coverage method 14

Supplementary Figure 5 | Inventory completeness of each taxonomic group estimated by the species accumulation curve method........ 15

Supplementary Figure 6 | Latitudinal patterns in species richness, spatial gaps and sampling efficiency for marine taxa under different spatial resolutions 16

Supplementary Figure 7 - 16 | Latitudinal patterns in species richness (observed and missing), number of sampling events, and estimates of inventory completeness of each taxonomic group at three different depth strata (euphotic, bathyal and abyssal)........ 17

Supplementary Figure 17 | Latitudinal pattern in species richness of each taxonomic group under standardized sample coverage 27

Supplementary Figure 18 | Latitudinal pattern in relative species richness under standardized sample size and coverage........ 28

Supplementary Figure 19 | Comparation between data from OBIS and from OBIS + additional nine datasets. 29

Supplementary Figure 20 | Latitudinal patterns in number of records and sampling events lacking species-level identification........ 30

Supplementary Figure 21 | Global distribution of all sampling events used in this study 31

Supplementary Figure 22 | Relationship between estimates of inventory completeness and number of unique records for each taxonomic group 32

Supplementary Table 1 | Number of records (total and valid) and relative proportion (%) of Ophiuroidea records from ten data repositories used in our study 34

Supplementary Table 2 | Pearson product-moment correlation coefficients between latitudinal patterns in observed ophiuroids species richness, number of absent species, number of sampling events and estimates of inventory completeness between OBIS dataset and OBIS + additional datasets.................................. 35

Supplementary Table 3 | Number of records of all taxonomic group after each step of the data cleaning process (columns).. 36

Supplementary Table 4 | Impact of removing records with double zero coordinates on estimates of species with spatial gaps 37

Supplementary References 38

Supplementary Note 1 | Latitudinal range of marine species

To evaluate the range contiguity assumption for marine species at the scale of our analysis (5° latitudinal bands), we quantified spatial gaps in the latitudinal distribution of relatively well-known species using range maps provided by the International Union for the Conservation of Nature (IUCN). The IUCN range maps are based not only on point-occurrence records, but also on expert knowledge of the biology of species and habitat preference. We measured latitudinal gaps in geographical distribution of all Scombridae, Elasmobranchii and coral species available in the IUCN repository (http://www.iucnredlist.org). Unfortunately, these are the only marine groups available in the IUCN repository among the taxonomic groups in our study.

We also evaluated the range cohesion of the marine species using estimates of species distribution models, which are less sensitive to geographical gaps in sampling effort and have been considered a good alternative to estimate complete species range[1]. All the modeled range maps were retrieved from AquaMaps (https://www.aquamaps.org), which provides standardized range maps for marine species based on environmental tolerances with respect to depth, salinity, temperature and primary production. A large part of species with range maps available in the IUCN repository also had estimated ranges in AquaMaps, except for corals (Scombridae: 98%, Elasmobranchii: 71%, Corals: 0.8%).

Our results show that range contiguity is the most common distribution pattern in nature (Supplementary Fig. 1). Only 10.7% of species have disjunct latitudinal range according to IUCN's range maps, whereas this estimate drops to only 2.7% according to species distribution models. Conversely, according to OBIS occurrence records, 57.3% have range discontinuities at 5° latitudinal resolution. It is also noteworthy that the equatorial dip in species diversity tends to decrease, or even disappear, when using range maps (Supplementary Fig. 1). Therefore, our results suggest that the geographical distribution of most marine species tends to be contiguous across space.

Supplementary Note 2 | Bathymetric distribution of marine species records

Records from relatively shallow waters (0-2,000 m) represent 89.23% of all records analysed in our study. Considering only the records with depth information and within our range of analysis (0 – 6,500 m), euphotic and bathyal strata represent 99% of all records (euphotic: 87.97%; bathyal: 11.10%), meaning that less than 1% of information came from the deep sea, the largest habitat on Earth. The maximum sampling effort per latitudinal band for any taxon, for example, decreased two orders of magnitude toward deeper strata (euphotic: 59,288; bathyal: 14,775; abyssal: 313). Consequently, the estimates of inventory completeness were, on average, lower at the deep sea than in shallow waters, especially in the southern hemisphere (see Fig. 3f in the main text). The total number of species, and species with gaps in their latitudinal range, also decreased 4.78 and 4.74 times, respectively, from euphotic to the deep-sea stratum (euphotic: 26,538 - 8,262; bathyal: 15,828 - 4,735; abyssal: 5,551 - 1,744).

Supplementary Note 3 | Standardizing sample size and completeness

Because tropical marine biota is undersampled, a direct comparation of species richness across latitudinal bands is not appropriated. However, two strategies may be employed to overcome this problem. First, it is possible to standardize the sample size by randomly subsampling the records across latitudinal bands to the same level as observed in the tropics. We repeated this procedure 1000 times and calculated the average latitudinal variation in species richness and completeness estimate expected in a scenario of homogeneous sampling effort across the globe. Note that we did not standardize the number of records, but the number of sampling events. Therefore, the number of records by sampling event was not altered. Polar latitudes were not subsampled because they already have sampling intensity below the tropical threshold.

Second, it is also possible to standardize the sampling coverage (interpolation)[2]. Using the *iNEXT* R package[3], we reduced the sampling effort across latitudes to the lowest completeness estimate observed in the tropics (between -15° and 15°). Additionally, we extrapolated tropical species richness. It is noteworthy that such extrapolation should be extended only to a doubling of the reference sample size. Beyond that level the extrapolation could create unreliable estimates. For this reason, we did not extrapolate the species richness for all latitudes until estimated total completeness. Instead, we used the extrapolation to achieve a higher standardized sampling coverage[2].

Our results show that standardization of sampling effort have a great impact on the latitudinal pattern in marine species richness. For most groups, when sampling effort have equal size, the number of species at those rich mid-latitudes drops to a value similar or lower than that recorded near the equator (Supplementary Fig. 2). Interestingly, if this reduced sampling effort was real, many species would not have been recorded at mid-latitudes. The reduction in sampling effort and species richness also altered the completeness estimate. However, the changes were more modest at higher latitudes, especially for sample coverage (Supplementary Fig. 4-5). This result suggests that while many tropical species are rare (few records), species at high latitudes are equally abundant and well represented, keeping completeness estimate relatively high even under reduced sampling effort. The latitudinal difference in completeness estimate despite standardized sample size also reinforces that a homogeneous sampling effort could produce a heterogeneous latitudinal gradient in inventory completeness. Likewise, heterogeneous sampling effort could also produce homogeneous inventory completeness. Therefore, species rich areas require more sampling effort to reach high completeness, especially if most species are rare or spatially restricted.

The results of coverage-based standardization also showed that species richness tends to be higher near the equator, for both interpolation and extrapolation scenarios (Supplementary Fig.

17-18). Indeed, for some groups, the extrapolated species richness was even higher in the tropical dip than around it (see for example Ophiuroidea, Amphipoda, and Porifera in Supplementary Fig. 17). Interestingly, the extrapolation also revealed that doubling the sample size and increasing the completeness estimate had greater impact on tropical species richness, but almost did not affect the diversity at higher latitudes. Our results suggest that species richness should increase rapidly with additional sampling effort in the tropics, but substantial effort would be necessary to reach the same level of completeness as currently existing at higher latitudes.

Supplementary Note 4 | OBIS representativeness and inventory completeness estimate

To evaluate if data retrieved from OBIS is representative of current knowledge of global patterns of marine biodiversity, we compiled ophiuroids data from additional nine data repositories worldwide. All retrieved data were submitted to the same quality control procedures described in the main text. OBIS records represented 57.81% of all valid records in the full dataset (Supplementary Table 1). We then calculated independently for each dataset the number of observed species (richness observed), number of absent species (spatial gaps), number of sampling events, and estimated inventory completeness for each 5° latitudinal bands.

Following Stropp *et al.*[4], we employed three methods to estimate inventory completeness. First, we applied Sousa-Baena *et al.*[5] estimation by generating tables of unique combinations of species name, coordinates and date of collection. For each latitudinal band we then calculated the total number of observed species (S_{obs}), and the number of observed species in only one (a) or two (b) sampling events. Sampling event is a unique combination of latitude, longitude and date of collection. Sousa-Baena *et al.*[5] estimation can be calculated by:

$$C_i = \frac{S_{obs\,i}}{S_{obs\,i} + \left(a_i^2 \,/\, 2b_i\right)}$$

where C_i is the inventory completeness for the latitudinal band *i*. Completeness estimate cannot be calculated when the parameter *b* is not found.

The second method to estimate inventory completeness uses sample coverage[2]. For each latitudinal band we quantified the total number of records (*n*), and number of species observed in only one (*f1*) or two (*f2*) sampling events. Sample coverage can be calculated by:

$$K_i = 1 - \frac{f_1}{n}\left[\frac{(n-1)f_1}{(n-1)f_1 + 2f_2}\right]$$

where K_i is the estimated inventory completeness of latitudinal band *i*. Both *C* and *K* ranges from zero to one, with one indicating a complete inventory.

Finally, we also estimated inventory completeness by the curvilinearity of smoothed species accumulation curves (SACs)[6,7]. Smoothed SACs were calculated by the 'exact' method (function 'specaccum' in the R package *vegan*[8]). The average slope of the last 10% of SACs obtained for each latitudinal band reflects the degree of curvilinearity, and was used to estimate the inventory completeness (r_i). A flat slope indicates saturation in the sampling (high inventory completeness), but produces r_i value close to zero. We calculated the 1-completeness estimate r_i to convert the values to a normalized scale from zero to one, in which one indicate high inventory completeness[4].

We used Pearson's product-moment correlation to evaluate the congruence of latitudinal gradients in species richness, spatial gaps and sampling effort estimated using only OBIS and the full dataset (OBIS + additional datasets). Our results revealed a strong correspondence between the two datasets (Supplementary Table 2), suggesting that additional datasets are not substantially different from OBIS (Supplementary Fig. 1). Estimates of inventory completeness are also not affected by the dataset, suggesting that OBIS can be used in isolation to estimate the latitudinal pattern in inventory completeness (Supplementary Table 2).

Estimates using Sousa-Baena *et al.*[5] method were not so correlated between the two datasets when compared to the other two completeness estimates. In addition, the monotonic

relationship between completeness estimate and number of records seems more unstable under Sousa-Baena *et al.*[5] method (Supplementary Fig. 22), suggesting that this method is more susceptible to artefactual values of C in latitudinal bands with small number of sampling records[4,5]. Because many latitudinal bands have few sampling records of some taxonomic groups, we abandoned Sousa-Baena *et al.*[5] method in all further analyses.

Supplementary Note 5 | Data cleaning procedures

Over half of our initial dataset retrieved from OBIS (and additional repositories for Ophiuroidea) was eliminated during the data cleaning processes (65.82%; Supplementary Table 3). One third (33.81%) of excluded records were duplicates, and a quarter (26.51%) lacked species-level identification. Although exclusion of duplicates has no effect on presence of latitudinal gaps in species distribution, the effect of excluding records with missing species identification requires further investigation. In fact, the predominance of such records in tropical waters could indicate that tropical species absence may be caused by lack of taxonomic expertise, instead of low sampling effort. To further investigate such possibility, we studied the latitudinal distribution of records lacking species-level identification. The data used in this analysis was retrieved on May 30 (2018), and is not identical to the dataset used in our main analyses, retrieved months earlier. Comparison between the two datasets shows an increase of 7.1% in the number of total records, but an equivalent proportion of records missing species-level identification (29.97%). Most records with unidentified species are located in well-sampled mid-latitudes (Supplementary Fig. 20). While equatorial data (between -5° and 5°) comprise only 2.49% of all records without identification, 29.4% of records lacking species-level identification are located between 50° and 60°. Thus, exclusion of records lacking species-level identification during the data cleaning process does not seem to cause spatial gaps in species' ranges.

Another potential cause of spatial gaps at low latitudes could be the removal of records with both coordinates equal to zero. Such records were eliminated because 0-0 location is probably auto-filled by computers when coordinate fields are left blank[9]. Because there are not many records at the 0-0 location (Supplementary Table 4), the removal of these records should have minimal impact for the latitudinal pattern of species absence. However, the 0-0 location is an actual marine location in the Atlantic Ocean (Gulf of Guinea) and some of the excluded records at this location could potentially represent accurate records. Thus, we identified all species with records at the 0-0 location, and then quantified the proportion of species with range gaps. We found that less than 1% of the species with spatial gaps had any excluded 0-0 location record (Supplementary Table 4). In addition, most of the records for benthic taxa indicate an unrealistic shallow depth. While the 0-0 location has an actual depth around 4,900 meters, most of the 0-0 records for benthic taxa indicate a depth between 8 and 14 meters, clearly indicating auto-filled and wrong geographical coordinates.

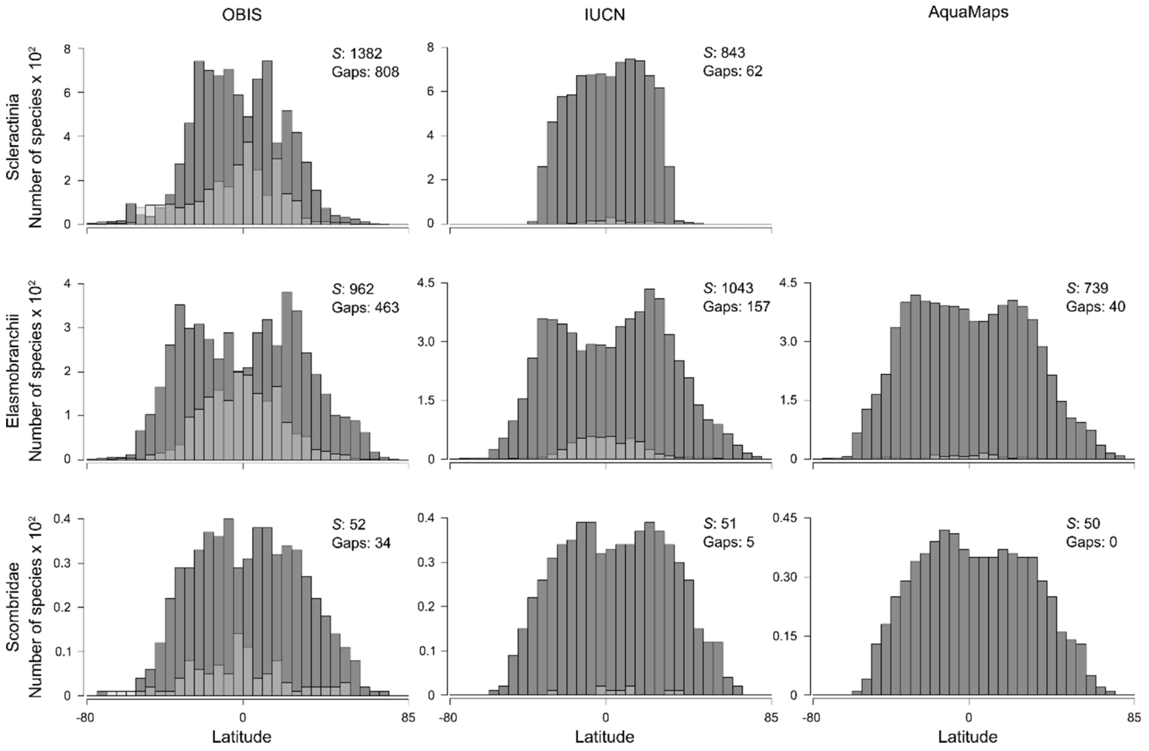

Supplementary Figure 1 | Latitudinal pattern in species richness according to different data sources. Dark grey bars represent number of observed species; light grey bars represent number of missing species (spatial gaps); colors are mixed where histograms overlap. S: total species richness; Gaps: number of species with any spatial gap. Notice that tropical peak in missing species coincides with the tropical dip in species richness. Range-based richness patterns (IUCN and AquaMaps) are not as bimodal as occurrence-based richness patterns (OBIS).

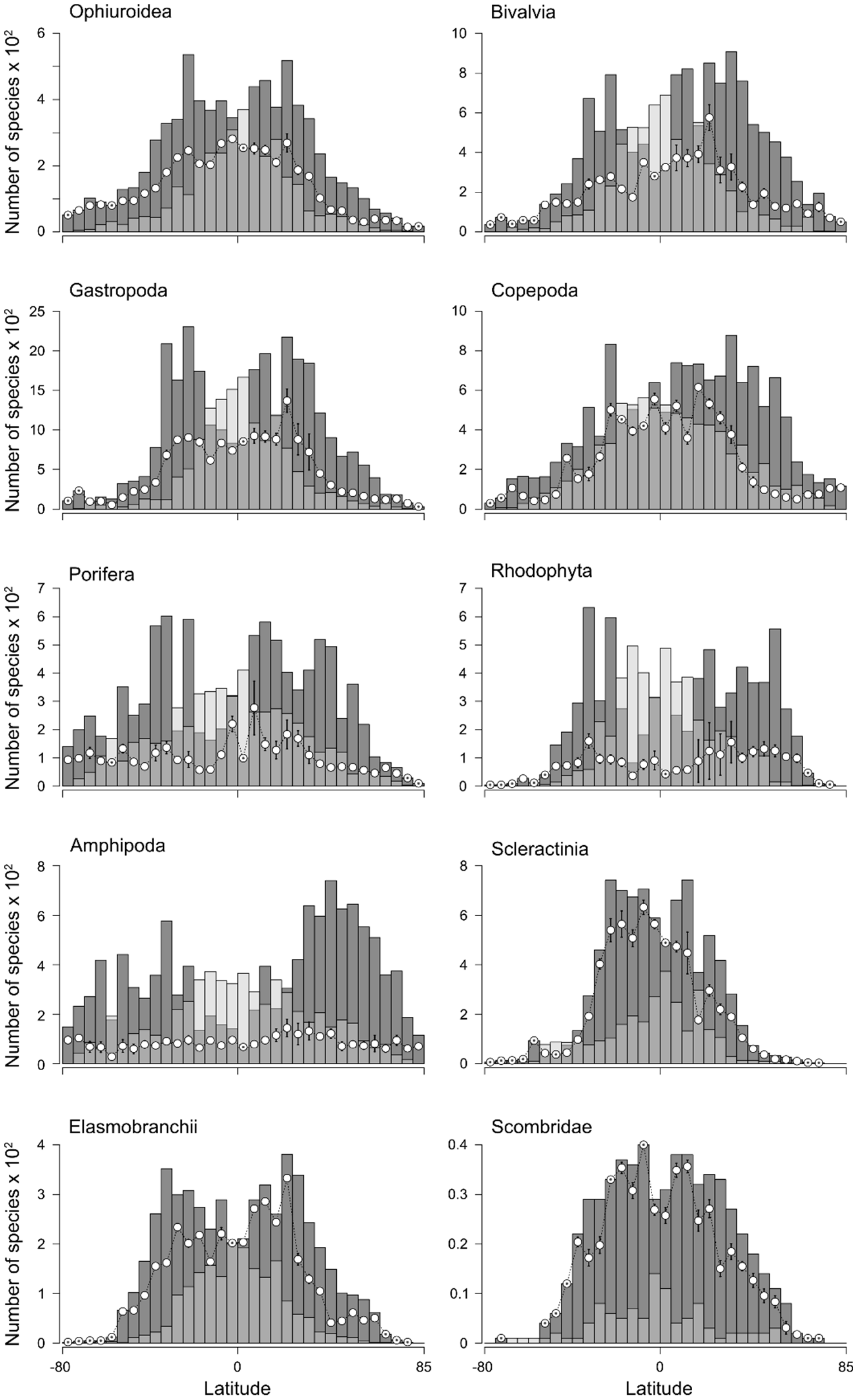

Supplementary Figure 2 | Latitudinal pattern in species richness of different taxonomic groups. Dark grey bars represent number of observed species; light grey bars represent number of missing species (spatial gaps); colors are mixed where histograms overlap. Circles represent mean (± s.d.) expected species richness under equally low sampling effort (see details in Supplementary Note 3). Filled circles indicate latitudes where sampling effort is already low and was not reduced. Notice that tropical peak in missing species coincides with the tropical dip in species richness.

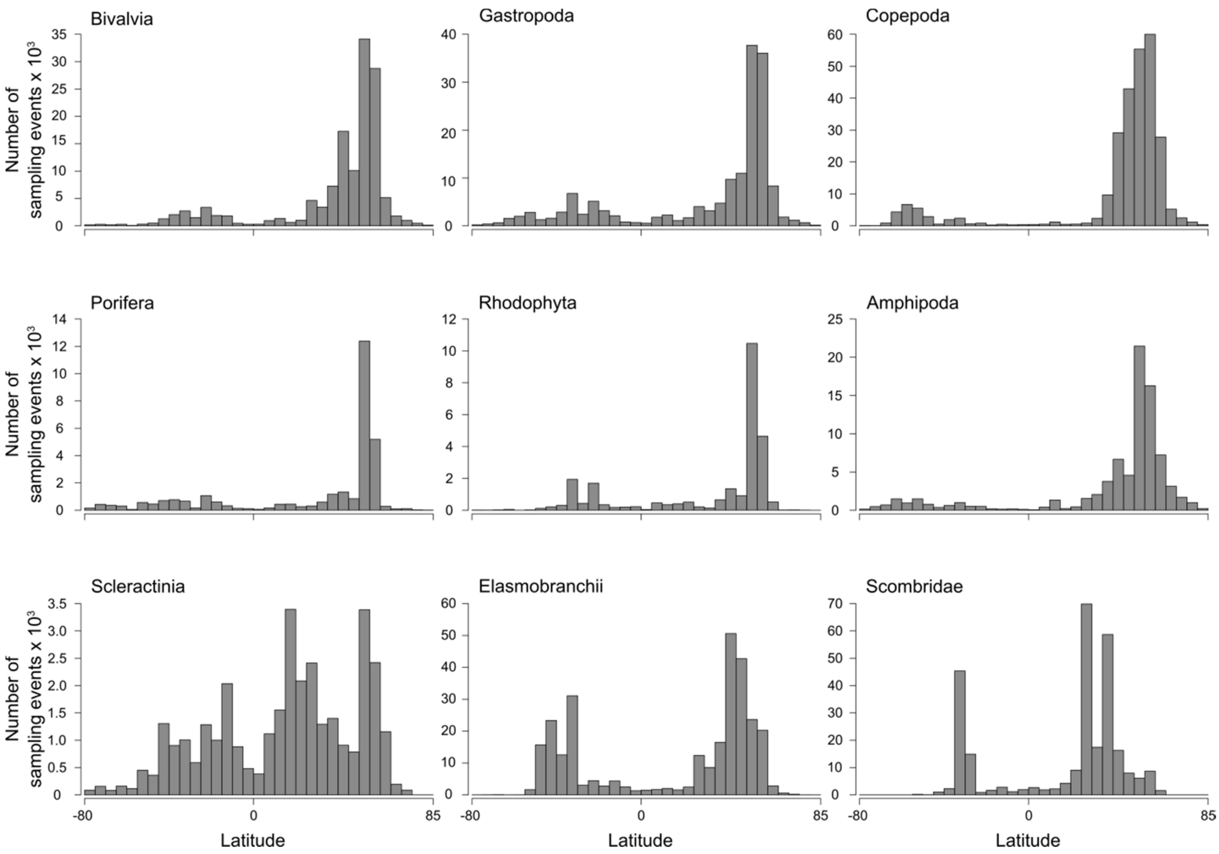

Supplementary Figure 3 | Latitudinal pattern in sampling events of each taxonomic group.

See Supplementary Fig. 19 for Ophiuroidea graph.

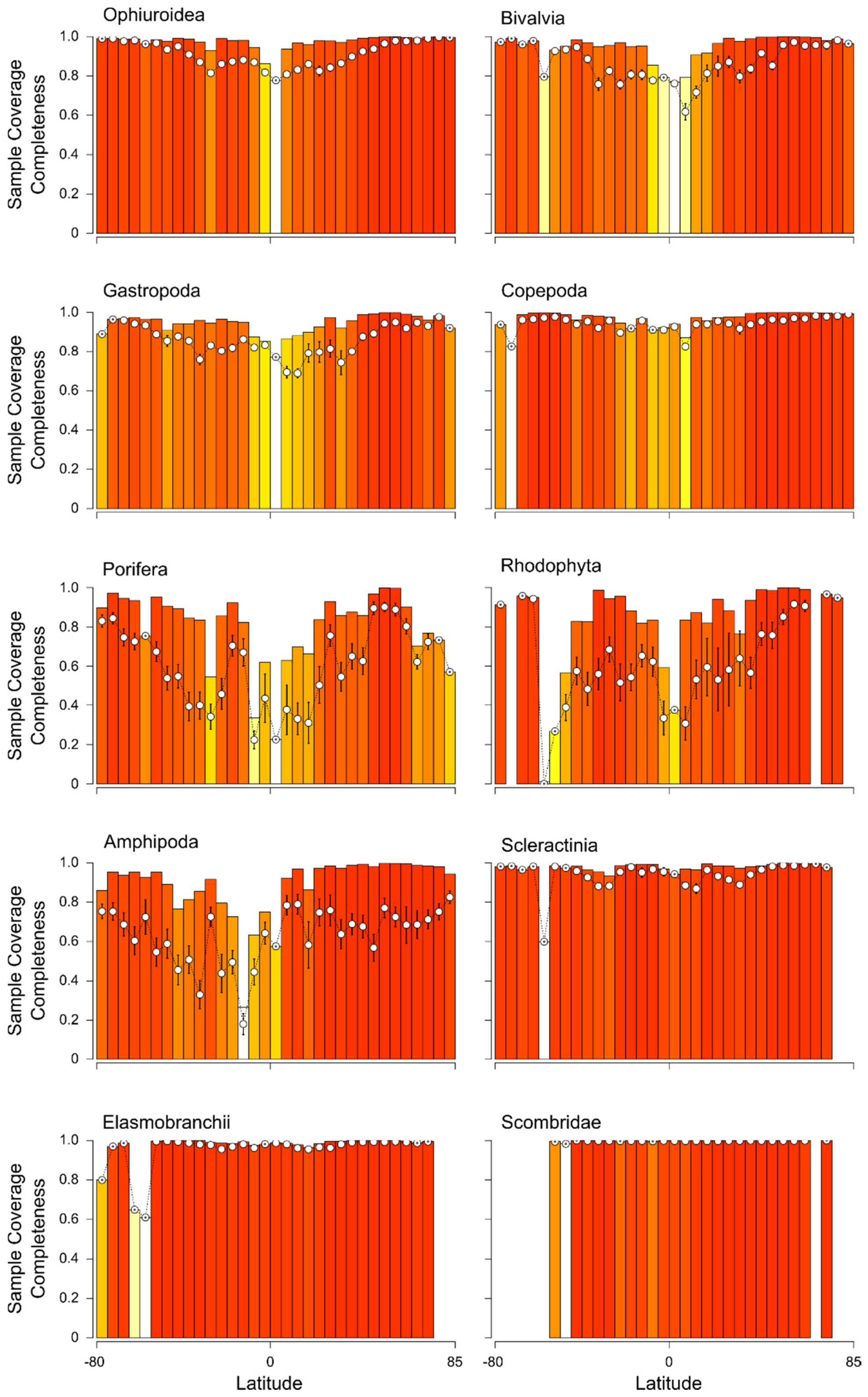

Supplementary Figure 4 | Inventory completeness of each taxonomic group estimated by the sample coverage method. Red indicates maximum and white indicates minimum estimated completeness level. Circles represent mean (± s.d.) expected inventory completeness under a scenario of homogeneous sampling effort across the globe (see details in Supplementary Note 3). Filled circles indicate poorly sampled latitudinal bands, in which subsampling was not applied.

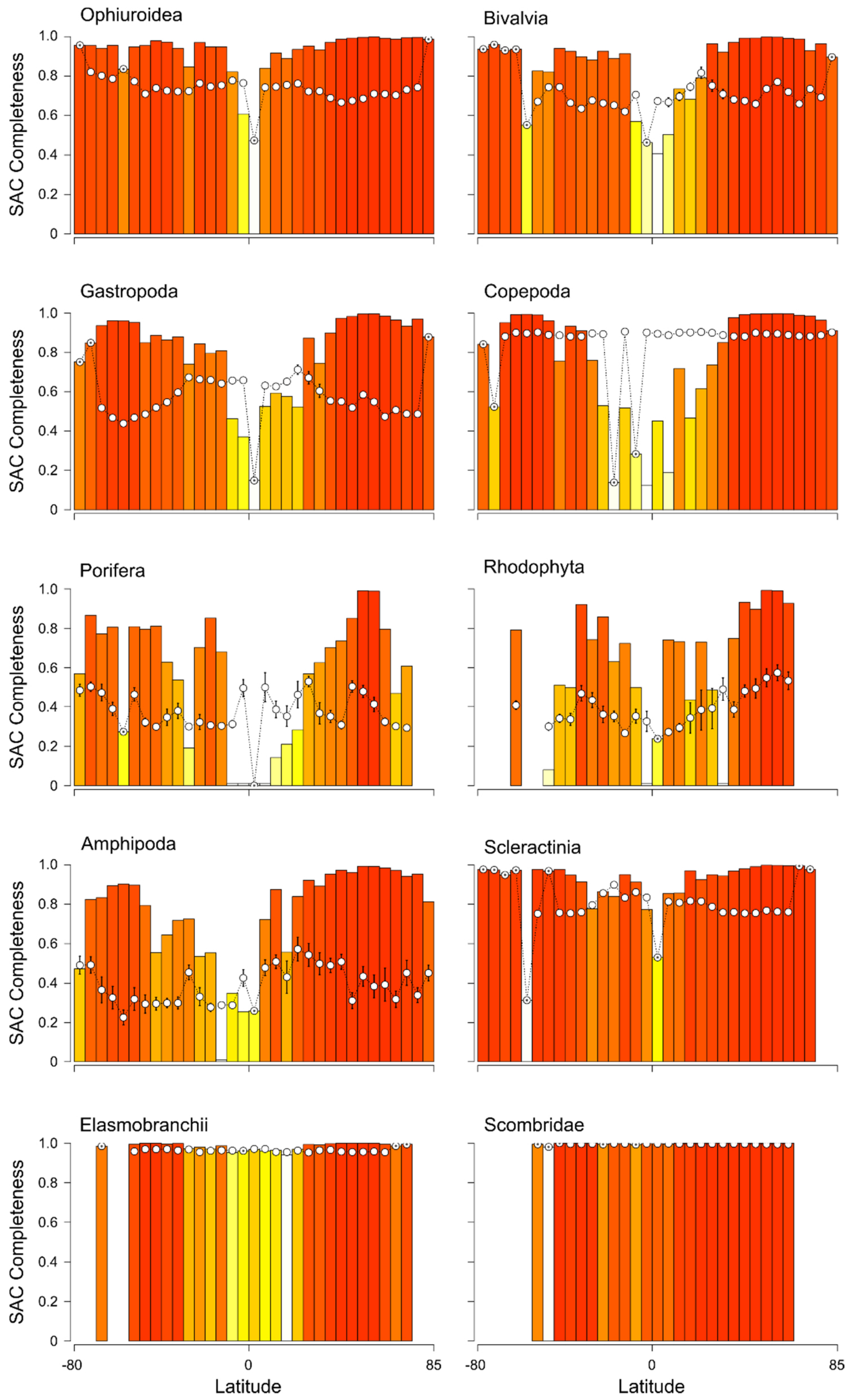

Supplementary Figure 5 | Inventory completeness of each taxonomic group estimated by the species accumulation curve method. See caption of Supplementary Fig. 4.

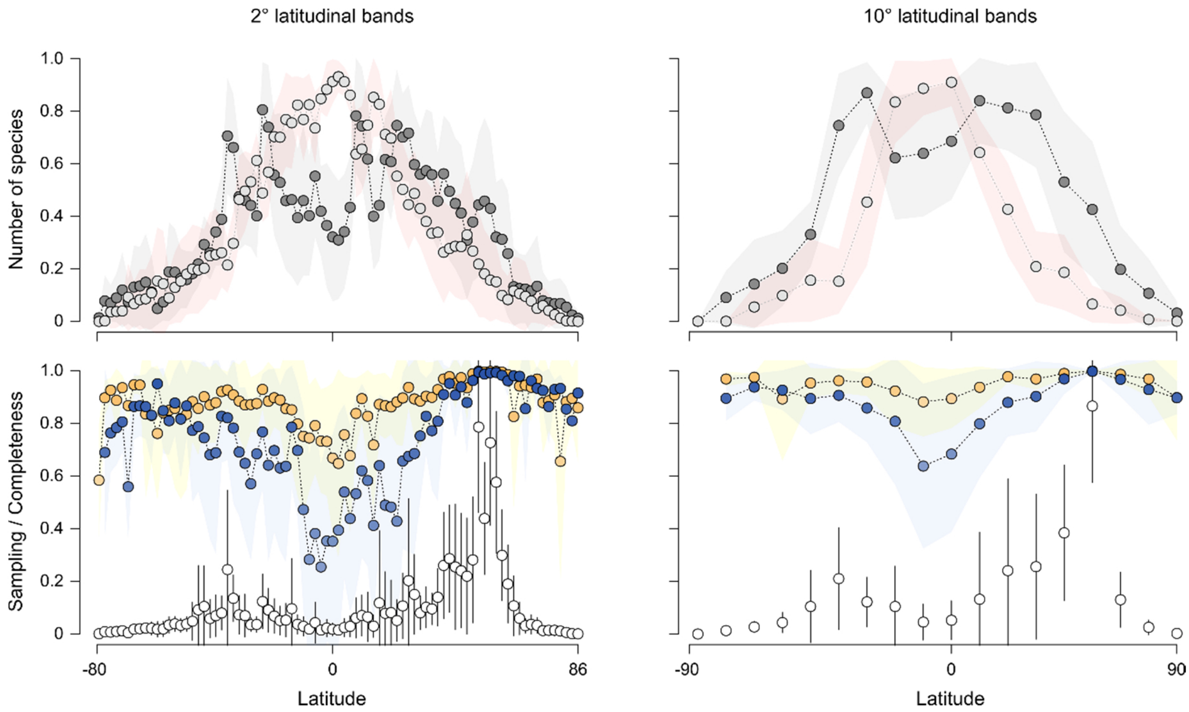

Supplementary Figure 6 | Latitudinal patterns in species richness, spatial gaps and sampling efficiency for marine taxa under different spatial resolutions. Circles represent mean values based on results of ten taxonomic groups. Shaded area and vertical bars represent standard deviation (s.d.). Data were standardized by the maximum observed value to range between 0 and 1 (except those which already vary on this scale). Top: Mean (± s.d.) latitudinal variation in number of observed species (dark grey circles) and number of missing species (light grey circles). Bottom: Mean (± s.d.) latitudinal variation in sampling effort (number of unique sampling events; open circles) and two estimates of inventory completeness (sample coverage: orange circles; species accumulation curve: dark blue circles).

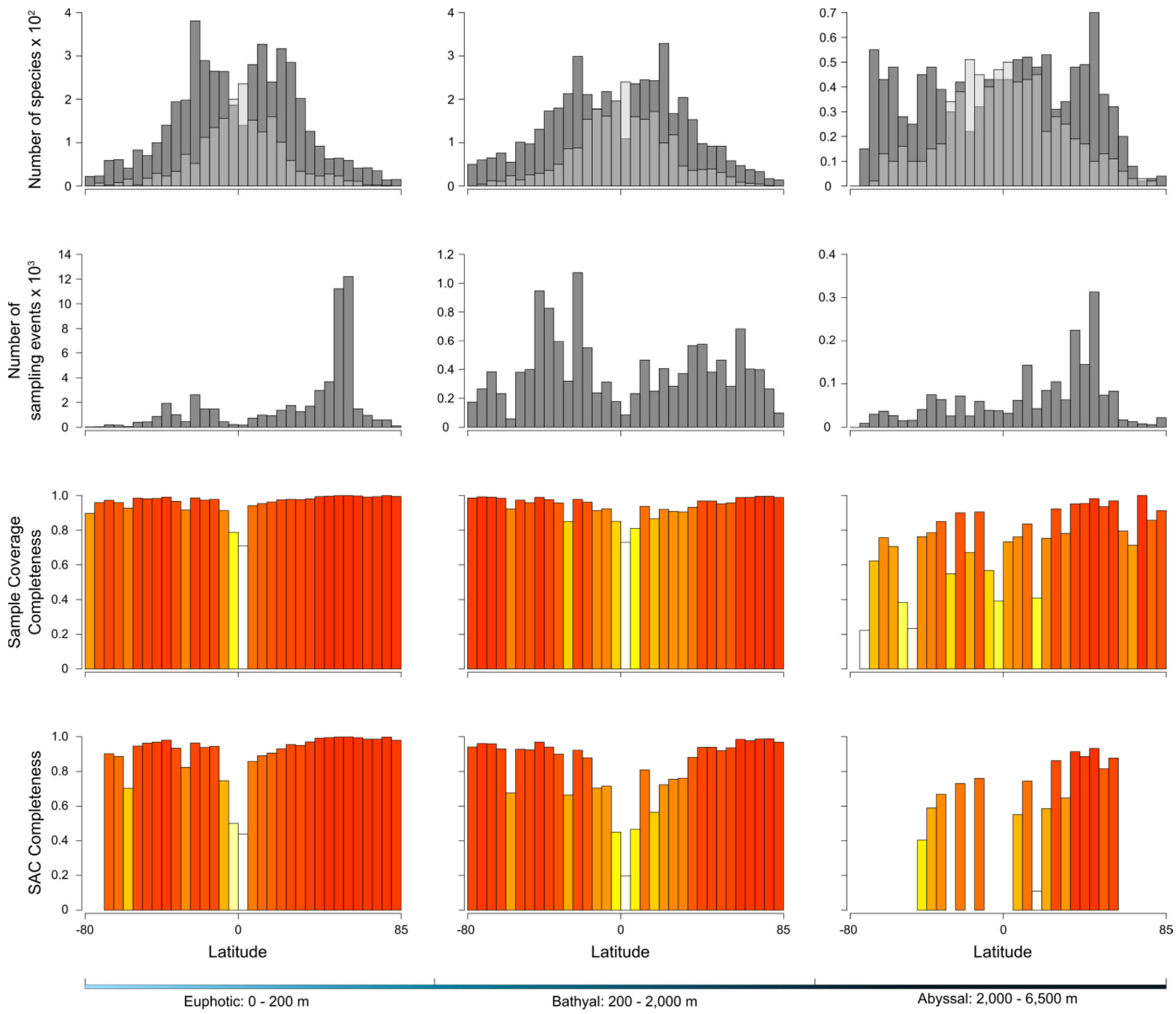

Supplementary Figure 7 | Latitudinal patterns in species richness (observed and missing), number of sampling events, and estimates of inventory completeness for Ophiuroidea at three different depth strata (euphotic, bathyal and abyssal). For species richness graphs, dark grey bars represent number of observed species; light grey bars represent number of missing species (spatial gaps); colors are mixed where histograms overlap. For inventory completeness, red indicates maximum and white indicates minimum level of completeness in the plot.

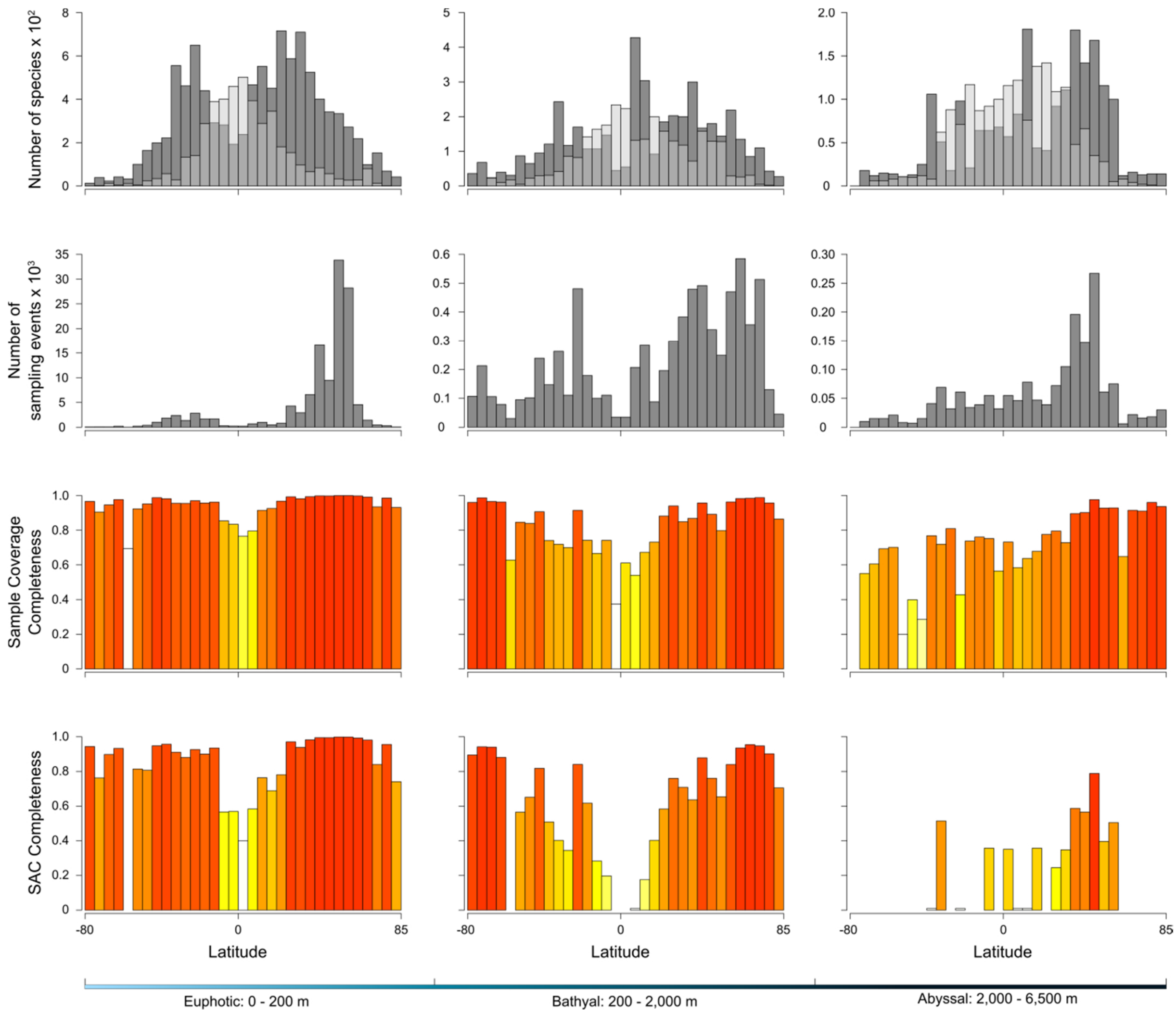

Supplementary Figure 8 | Latitudinal patterns in species richness (observed and missing), number of sampling events, and estimates of inventory completeness for Bivalvia at three different depth strata (euphotic, bathyal and abyssal). See caption of Supplementary Figure 7.

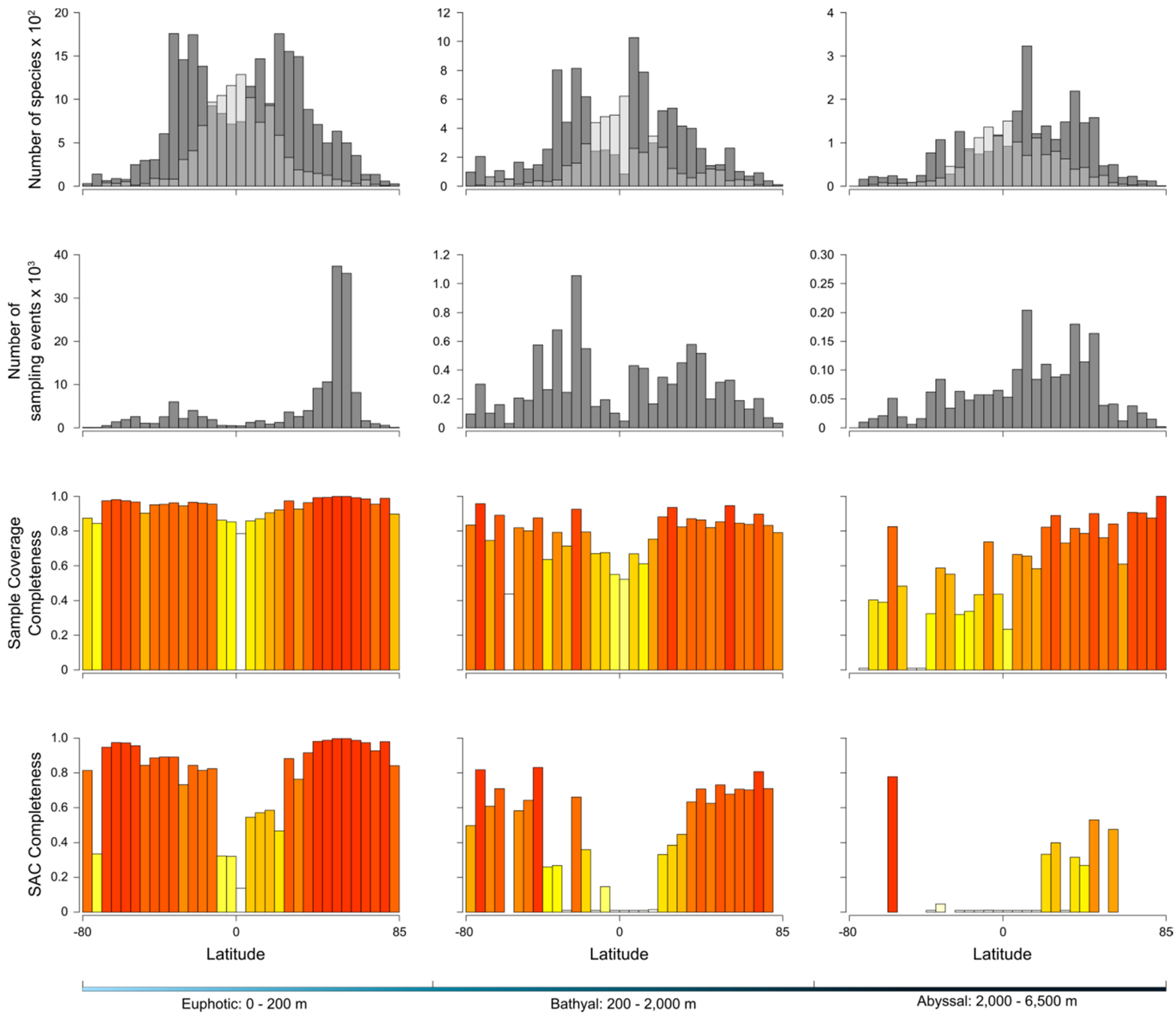

Supplementary Figure 9 | Latitudinal patterns in species richness (observed and missing), number of sampling events, and estimates of inventory completeness for Gastropoda at three different depth strata (euphotic, bathyal and abyssal). See caption of Supplementary Figure 7.

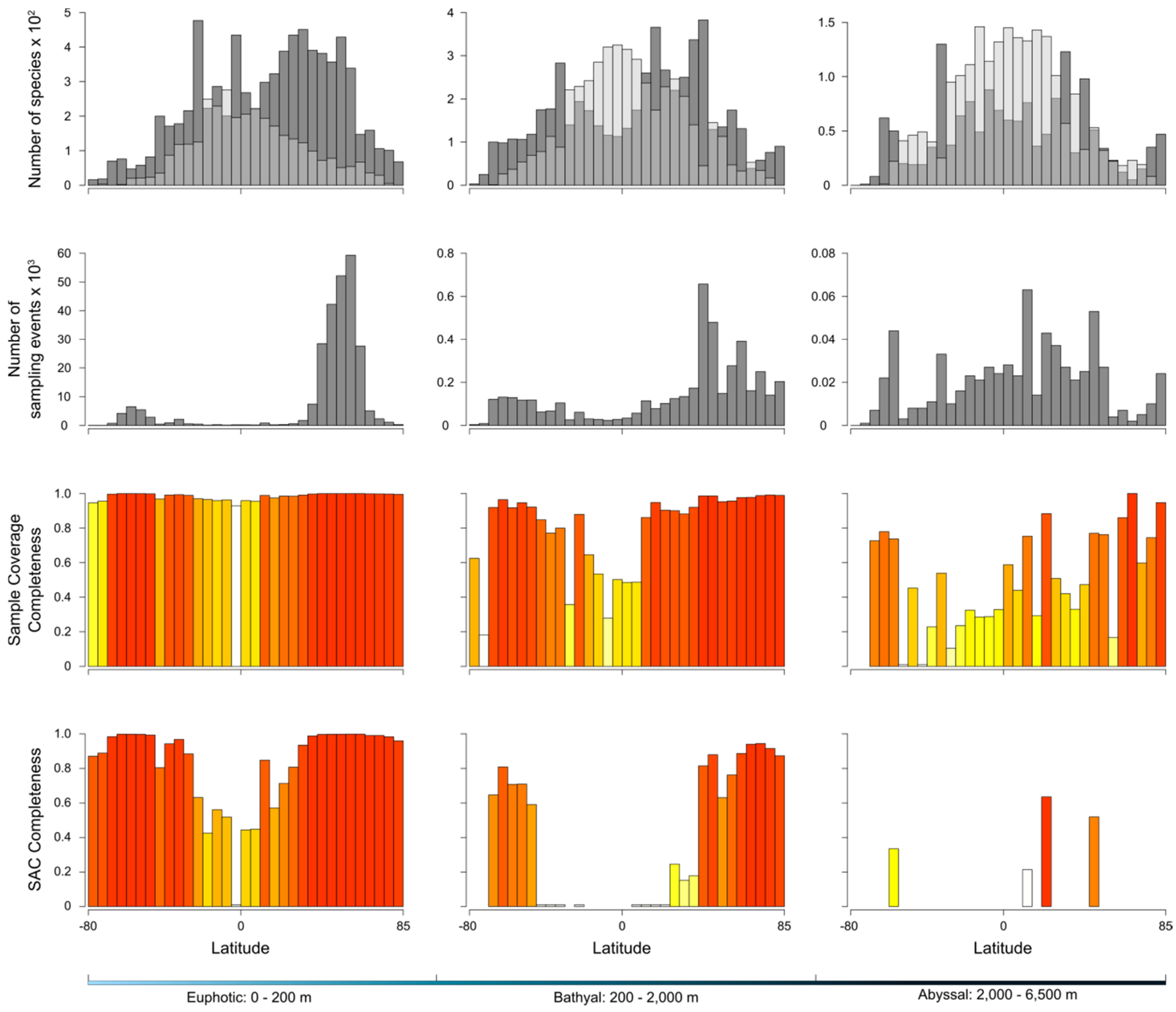

Supplementary Figure 10 | Latitudinal patterns in species richness (observed and missing), number of sampling events, and estimates of inventory completeness for Copepoda at three different depth strata (euphotic, bathyal and abyssal). See caption of Supplementary Figure 7.

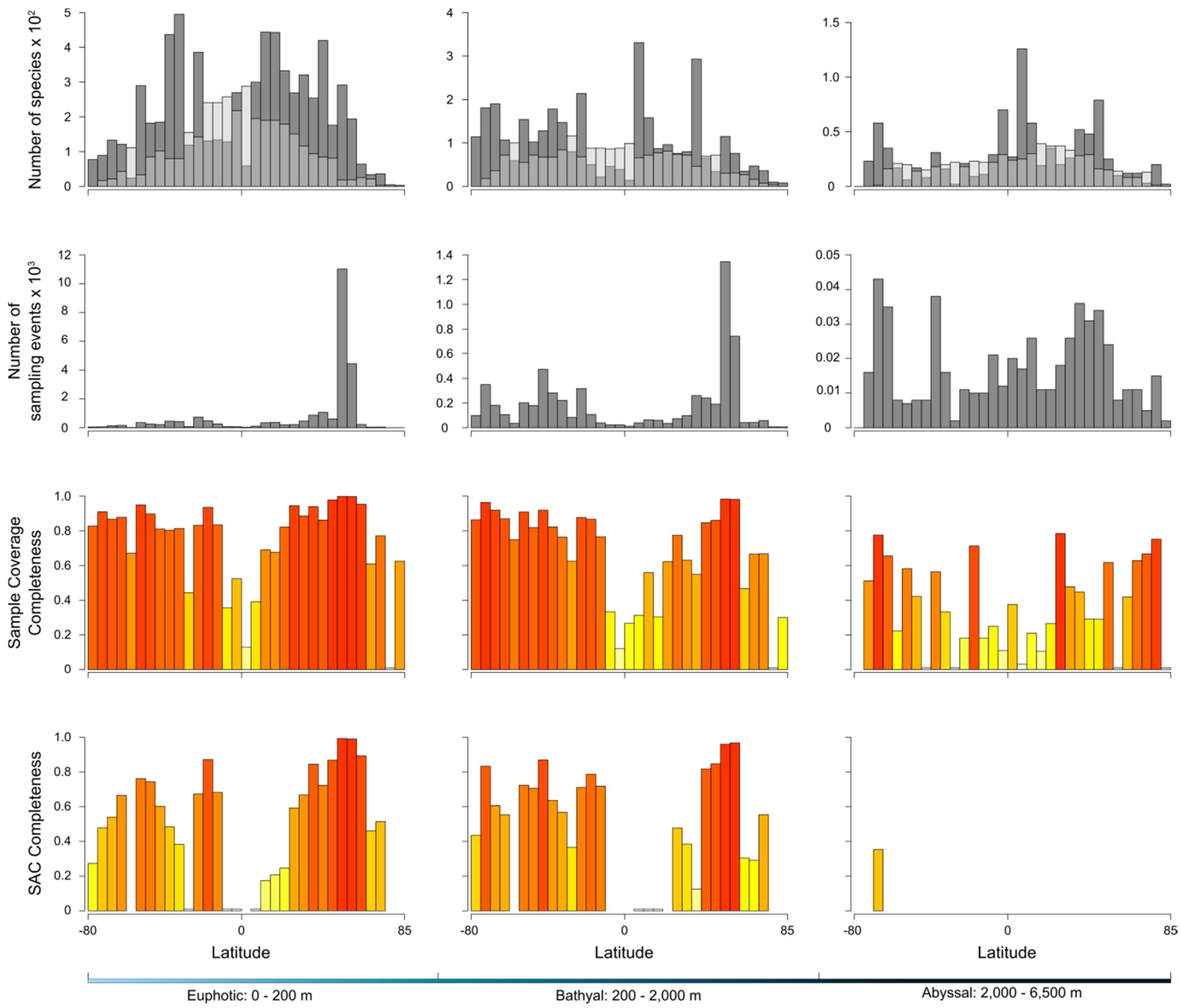

Supplementary Figure 11 | Latitudinal patterns in species richness (observed and missing), number of sampling events, and estimates of inventory completeness for Porifera at three different depth strata (euphotic, bathyal and abyssal). See caption of Supplementary Figure 7.

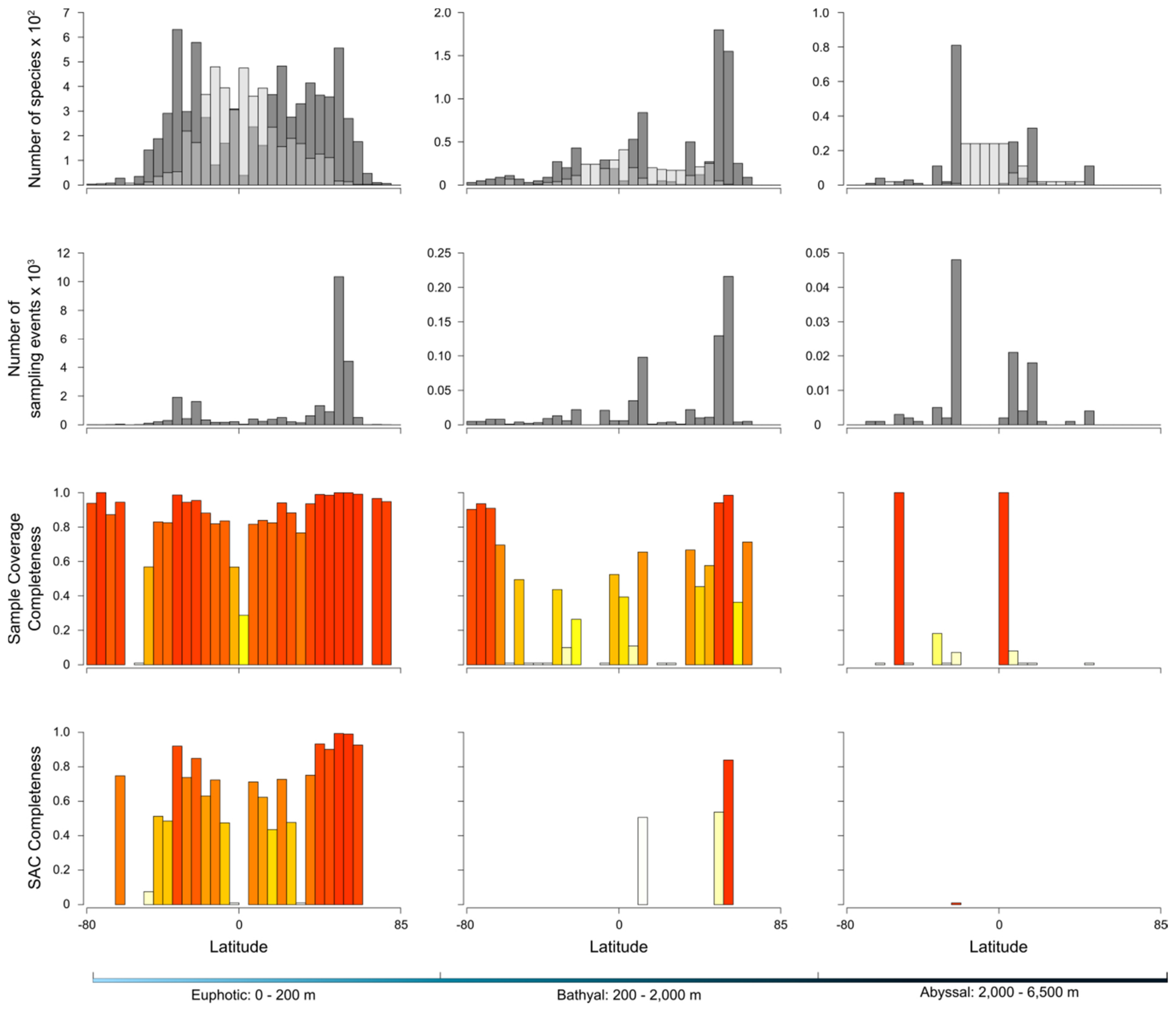

Supplementary Figure 12 | Latitudinal patterns in species richness (observed and missing), number of sampling events, and estimates of inventory completeness for Rhodophyta at three different depth strata (euphotic, bathyal and abyssal). See caption of Supplementary Figure 7.

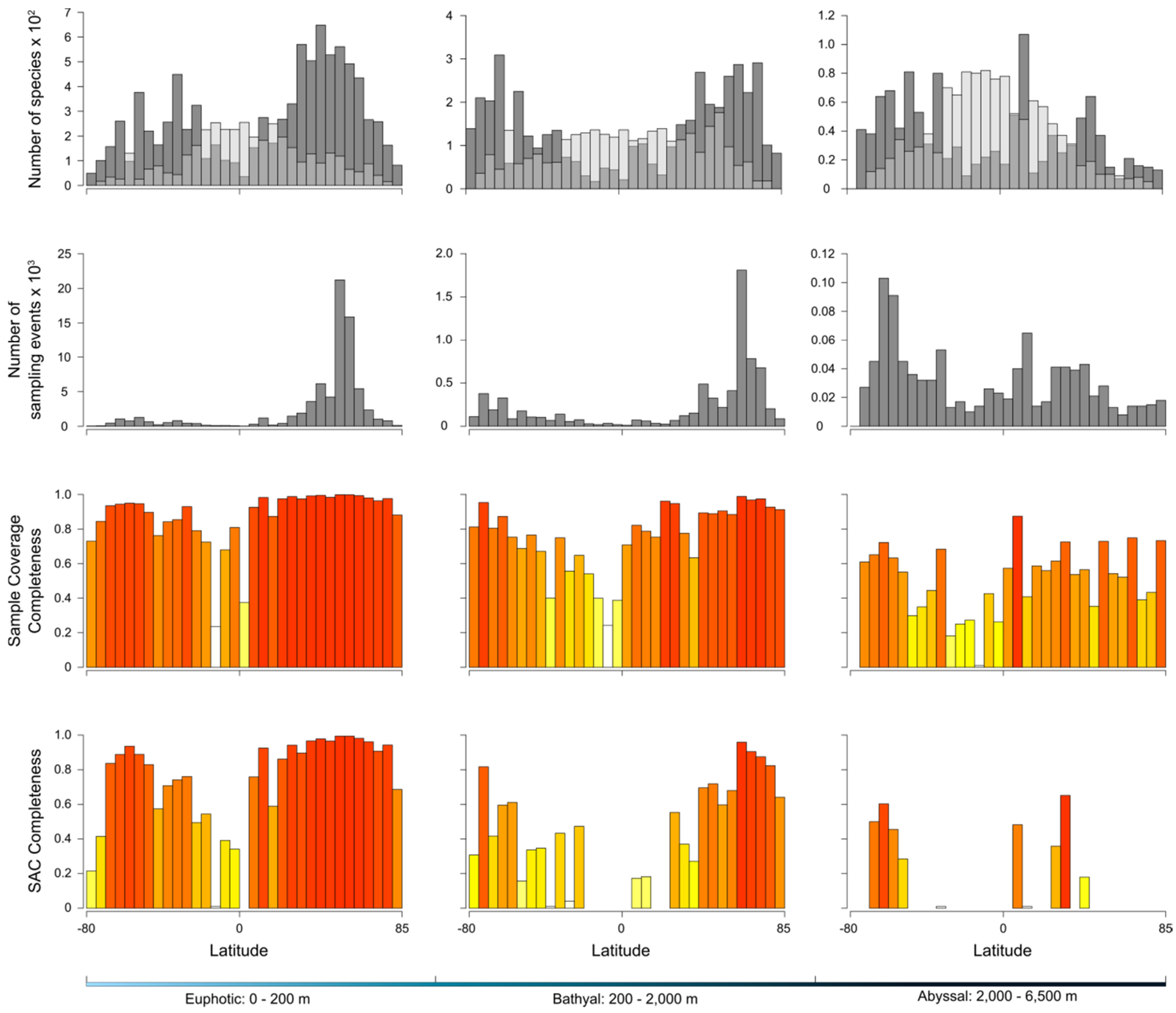

Supplementary Figure 13 | Latitudinal patterns in species richness (observed and missing), number of sampling events, and estimates of inventory completeness for Amphipoda at three different depth strata (euphotic, bathyal and abyssal). See caption of Supplementary Figure 7.

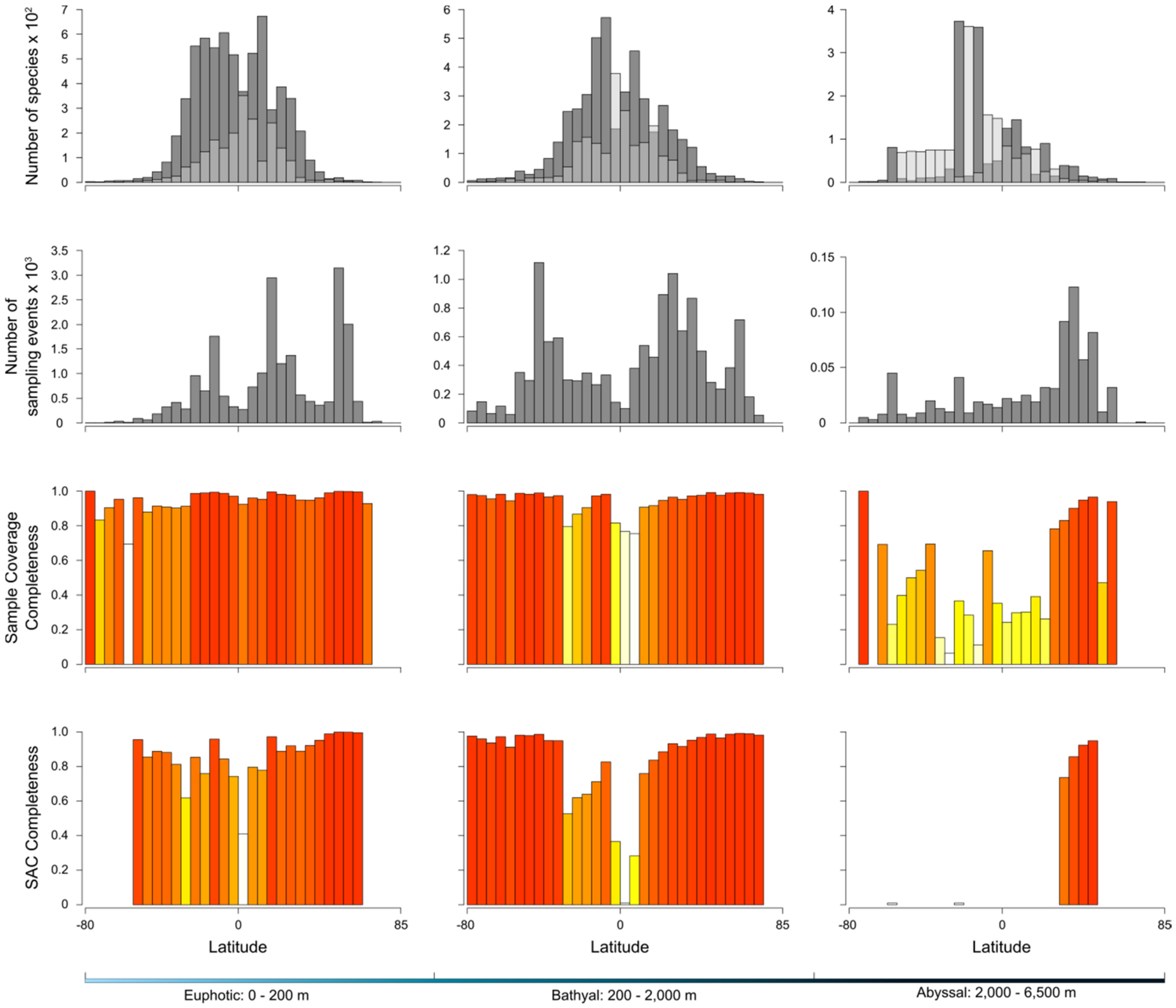

Supplementary Figure 14 | Latitudinal patterns in species richness (observed and missing), number of sampling events, and estimates of inventory completeness for Scleractinia at three different depth strata (euphotic, bathyal and abyssal). See caption of Supplementary Figure 7.

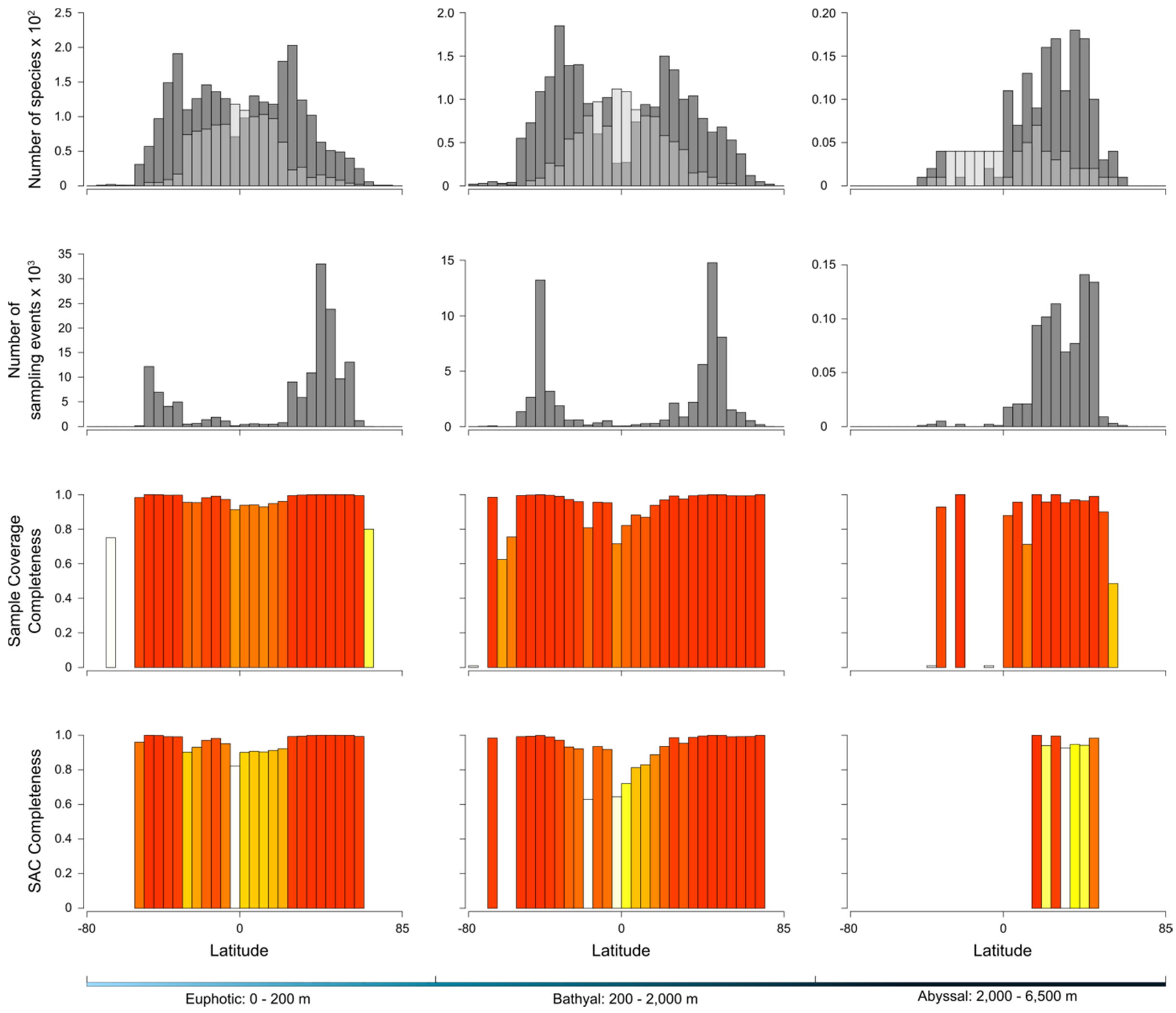

Supplementary Figure 15 | Latitudinal patterns in species richness (observed and missing), number of sampling events, and estimates of inventory completeness for Elasmobranchii at three different depth strata (euphotic, bathyal and abyssal). See caption of Supplementary Figure 7.

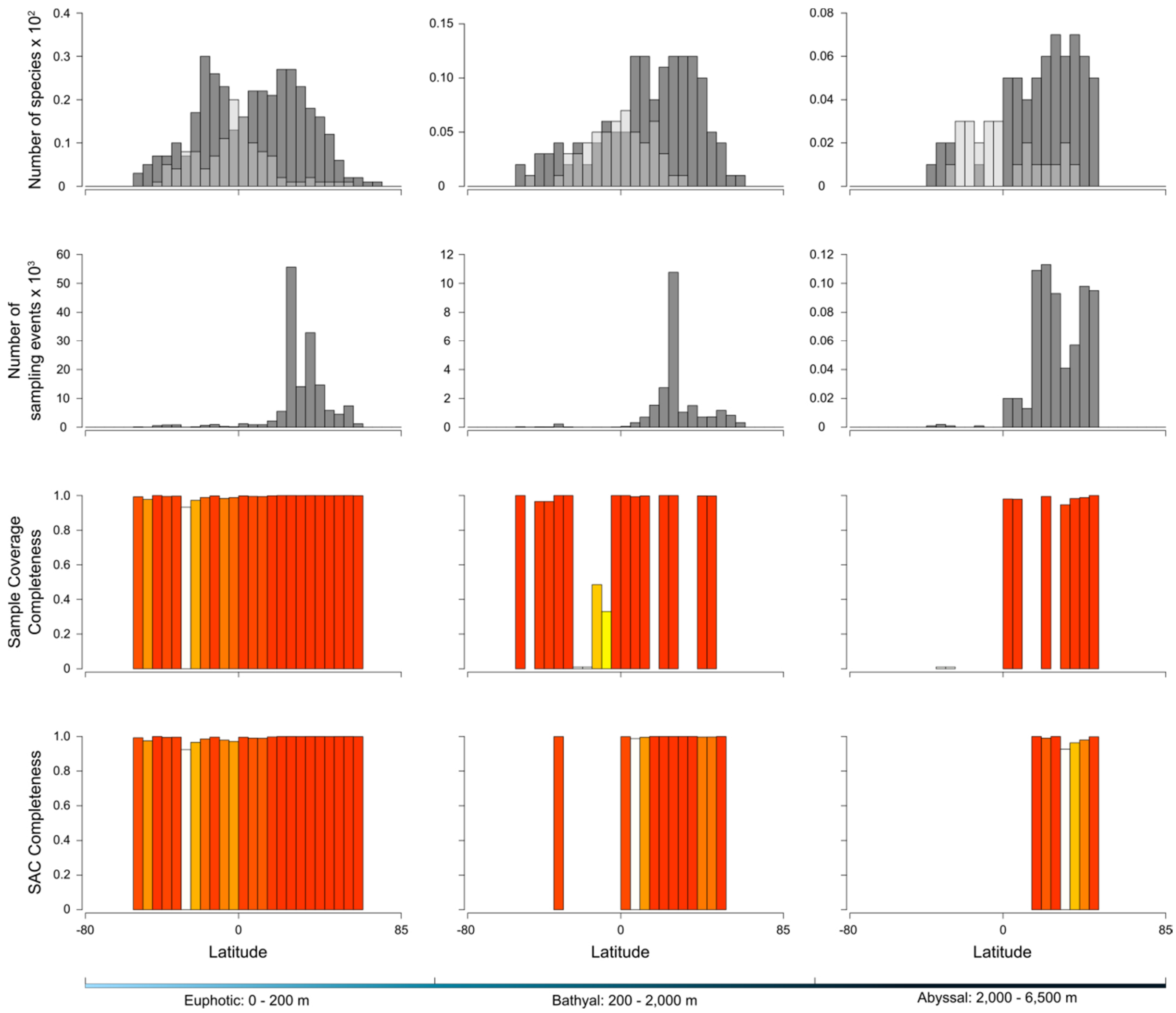

Supplementary Figure 16 | Latitudinal patterns in species richness (observed and missing), number of sampling events, and estimates of inventory completeness for Scombridae at three different depth strata (euphotic, bathyal and abyssal). See caption of Supplementary Figure 7.

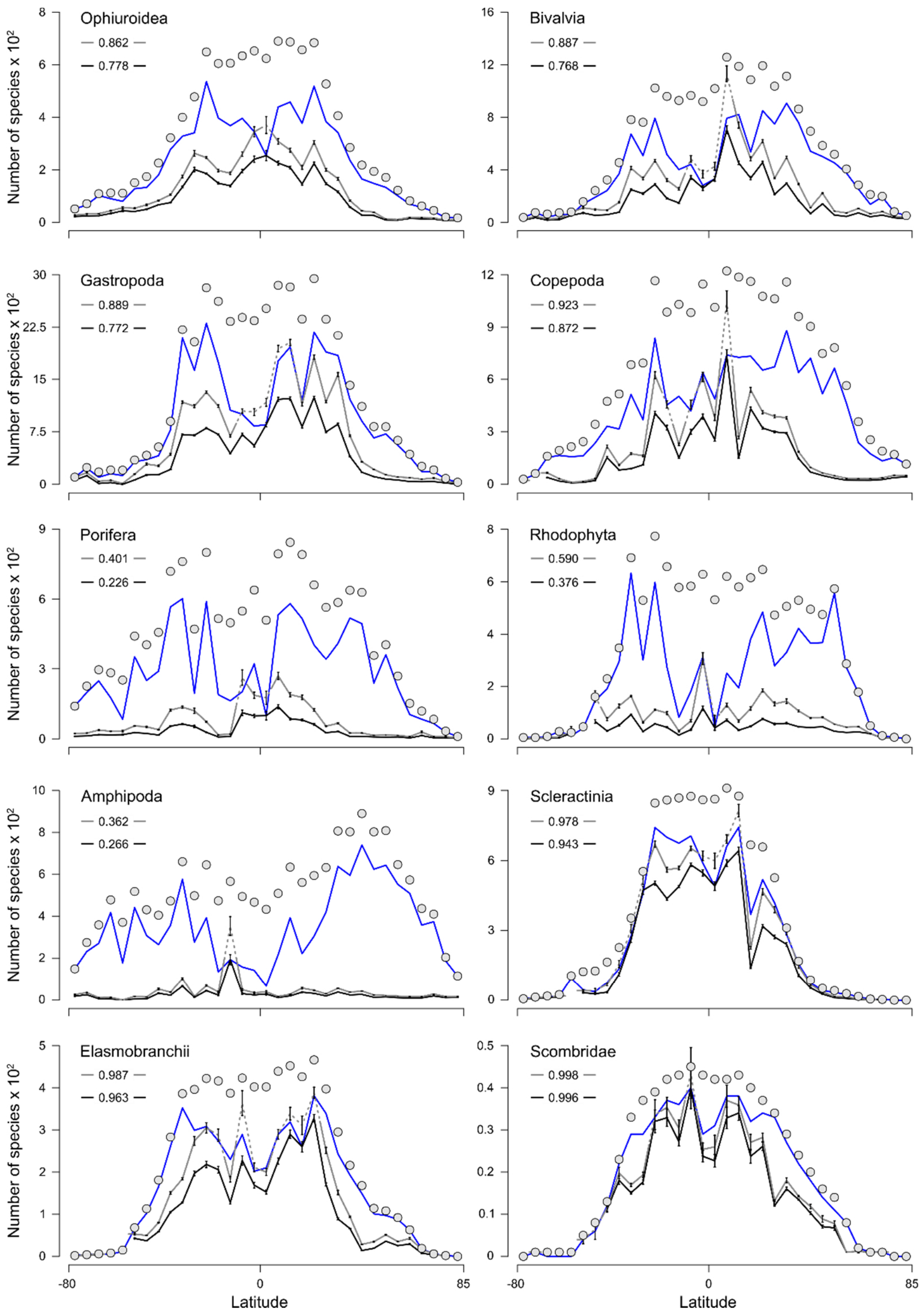

Supplementary Figure 17 | Latitudinal pattern in species richness of each taxonomic group under standardized sample coverage. Blue lines represent observed species richness; black lines represent expected species richness using only interpolation; grey lines represent expected species richness using interpolation (solid) and extrapolation (dotted). Error bars: 95% confidence interval. Numbers associated with lines (legend) indicate completeness level used to interpolate/extrapolate species richness. Grey circles represent expected species richness after accounting for spatial gaps in species distributions.

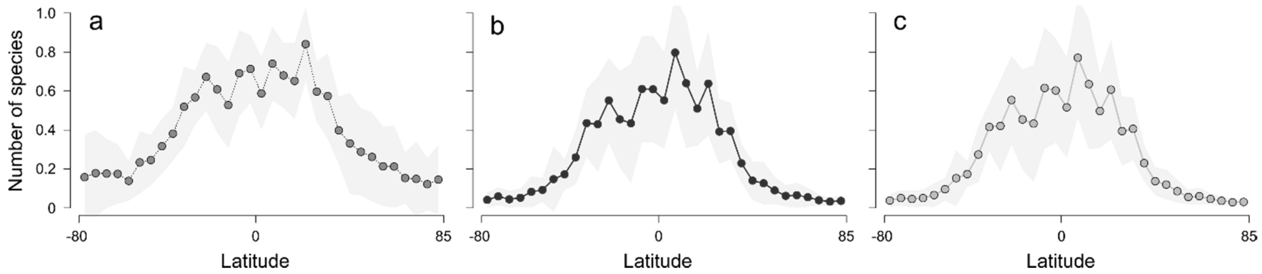

Supplementary Figure 18 | Latitudinal pattern in relative species richness under standardized sample size and coverage. Circles and shading represent mean and standard deviation of relative species richness of ten taxonomic groups. Data were standardized by the maximum observed value to range between 0 and 1. **(a)** Equal sample size. **(b)** Equal sampling coverage using only interpolation. **(c)** Equal sampling coverage using interpolation and extrapolation.

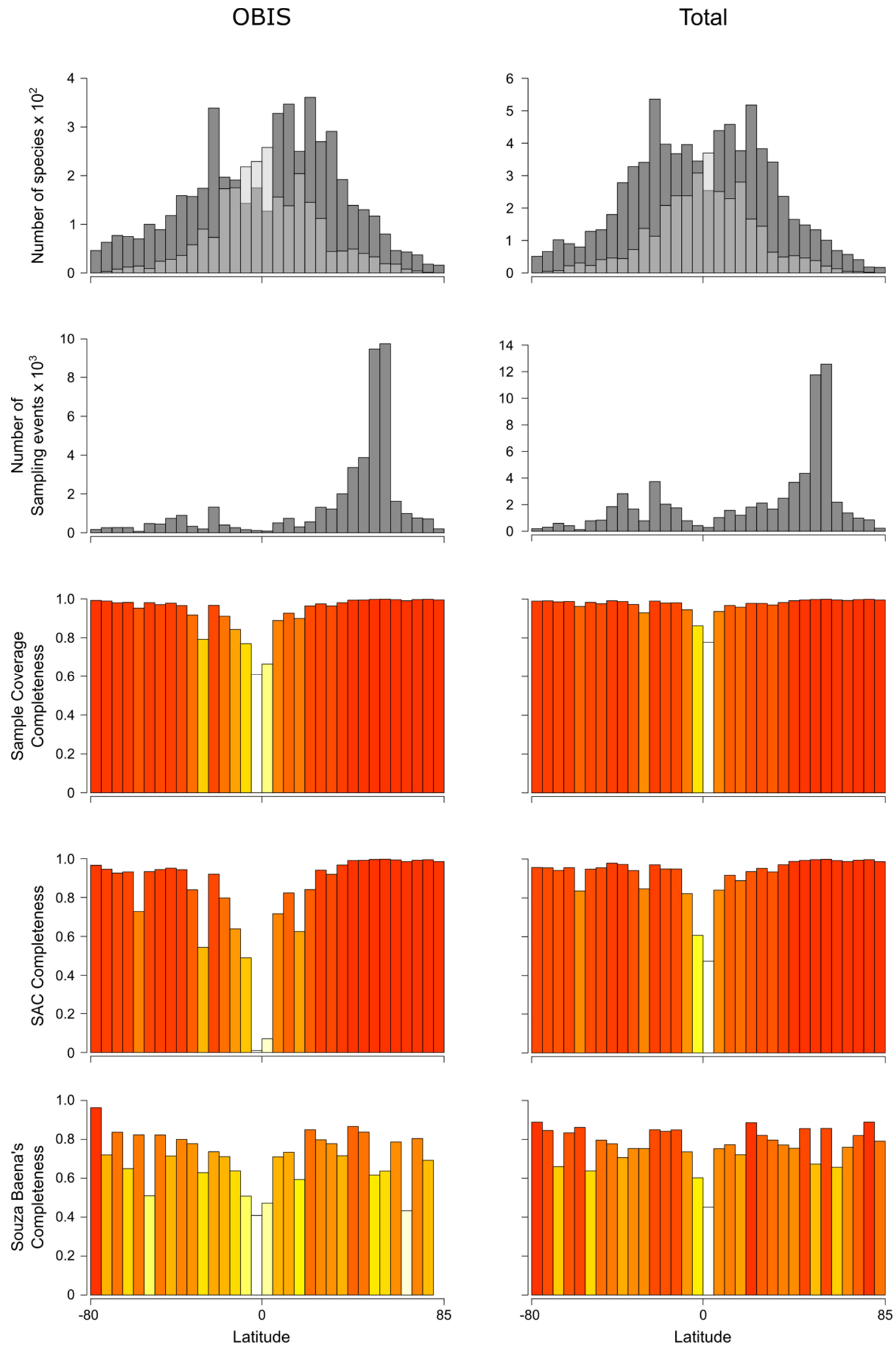

Supplementary Figure 19 | Comparation between data from OBIS and from OBIS + additional nine datasets. Latitudinal patterns in species richness (observed: dark grey; missing: light grey), number of sampling events, and estimates of inventory completeness for Ophiuroidea considering only data from OBIS (left) and from additional nine data repositories (right). See caption of Supplementary Figure 7.

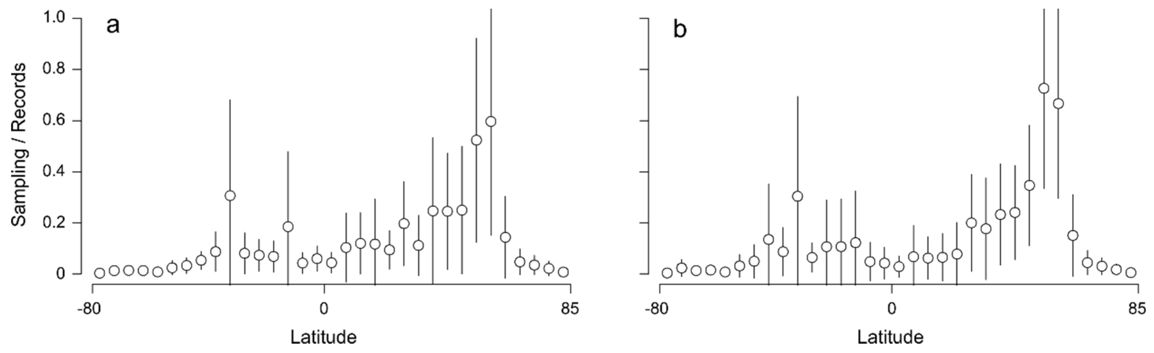

Supplementary Figure 20 | Latitudinal patterns in number of records and sampling events lacking species-level identification. Circles and vertical bars represent mean and standard deviation of ten taxonomic groups. Data were standardized by the maximum observed value to range between 0 and 1. **(a)** Total number of records. **(b)** Number of unique sampling events.

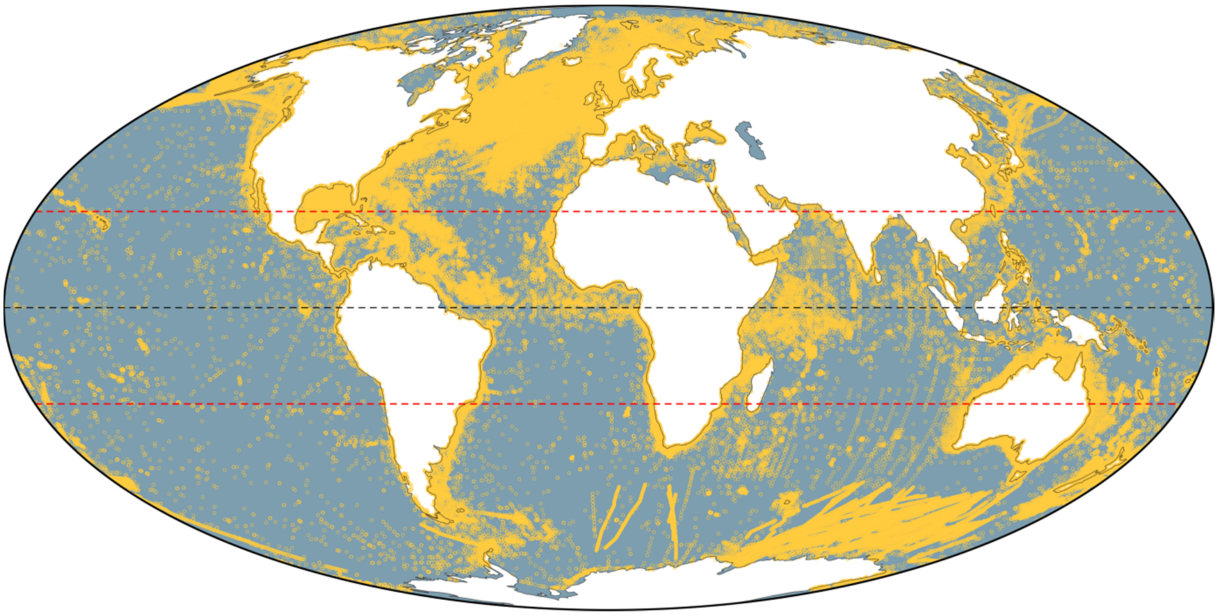

Supplementary Figure 21 | Global distribution of all sampling events used in this study. Land map used in this figure is in public domain, freely available from Natural Earth. See https://www.naturalearthdata.com/about/terms-of-use/ for more details.

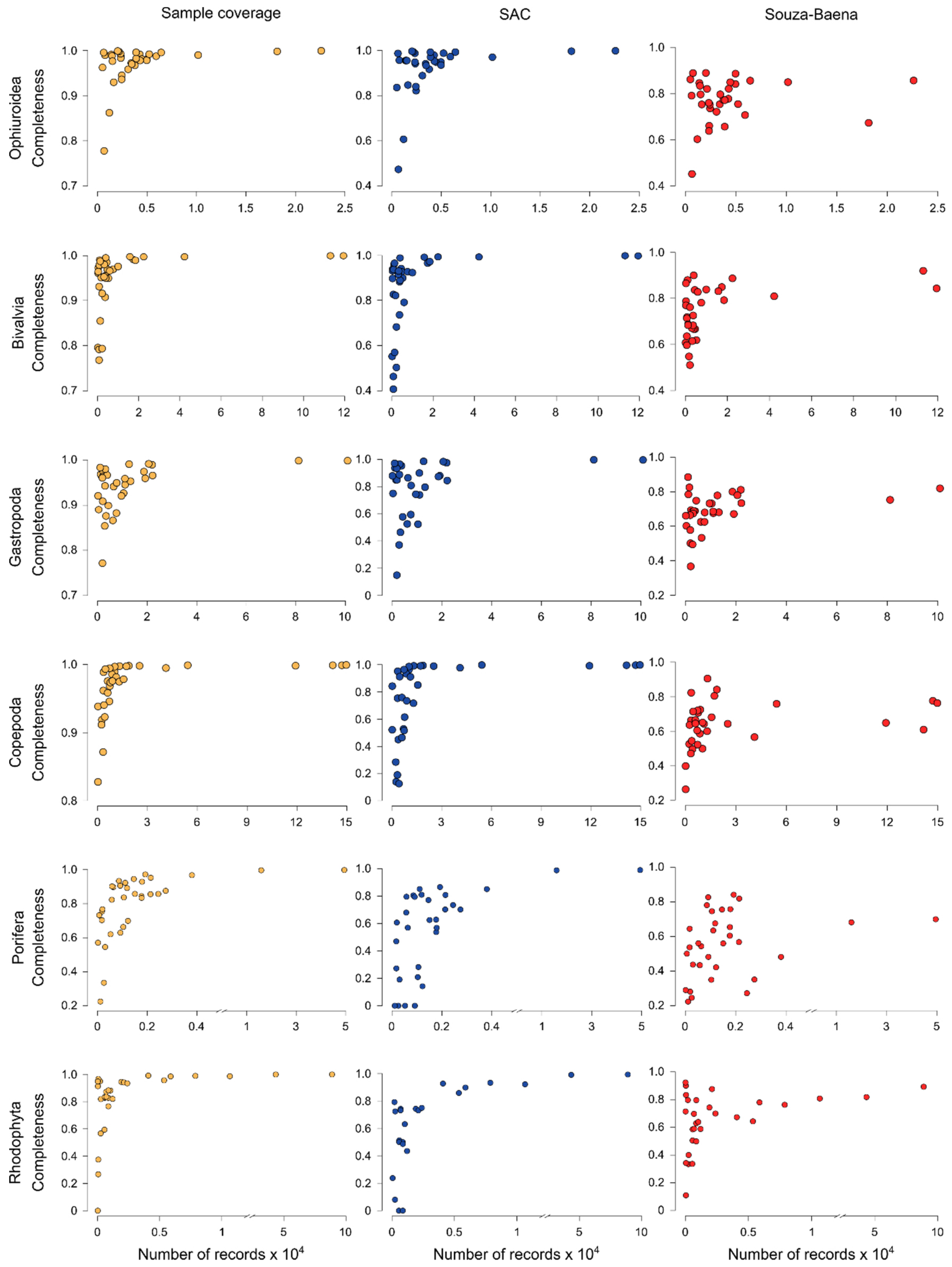

Supplementary Figure 22 | Relationship between estimates of inventory completeness and number of unique records for each taxonomic group. Points are different latitudinal bands. Notice the break in x-axis of Porifera and Rhodophyta plots. Figure continues to next page.

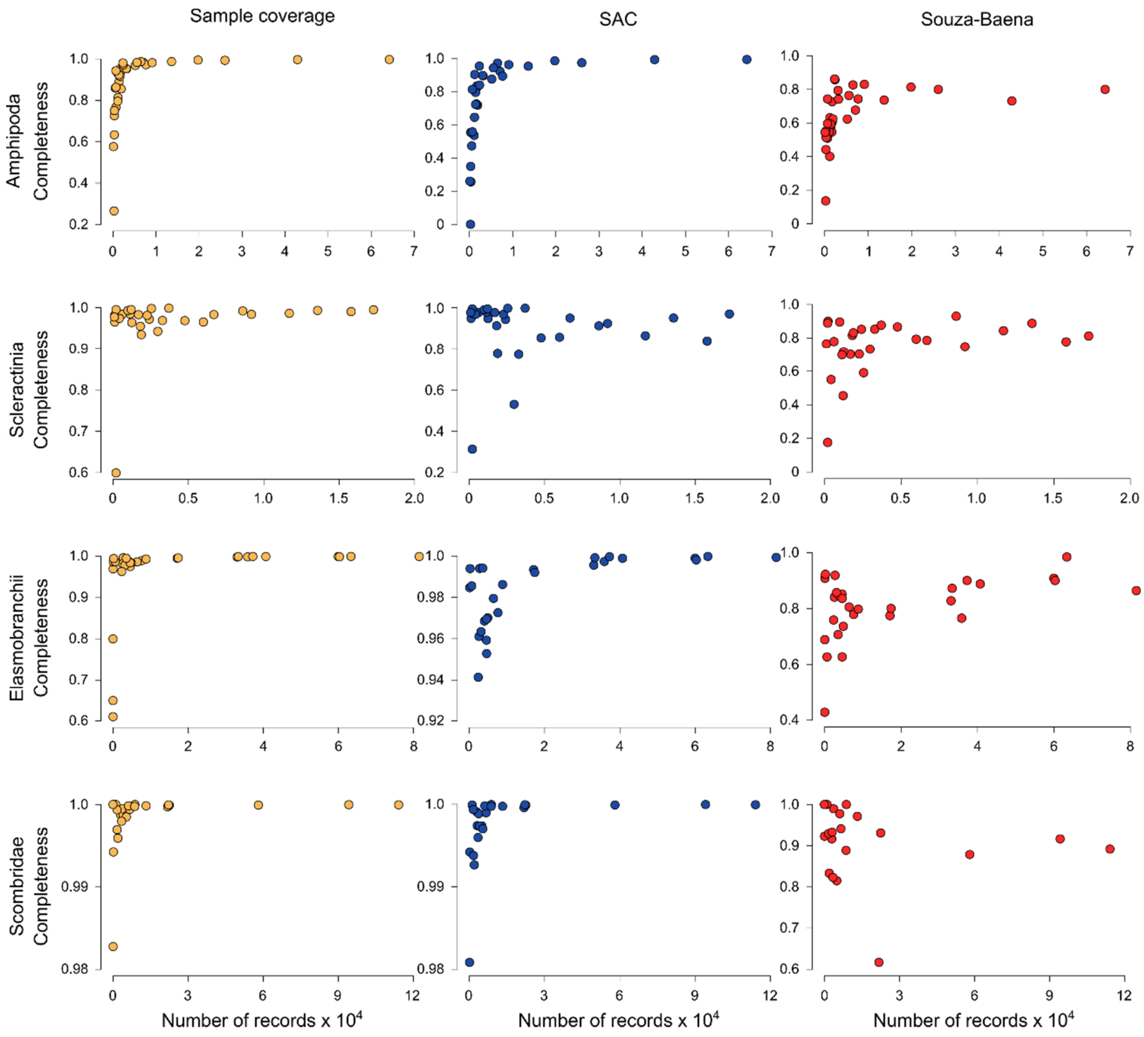

Supplementary Figure 22 | Continued.

Supplementary Table 1 | Number of records (total and valid) and relative proportion (%) of Ophiuroidea records from ten data repositories used in our study. VR are records kept after data cleaning procedures. OBIS - Ocean Biogeographic Information System; GBIF - Global Biodiversity Information Facility; O'Hara *et al.* - reference in the main text; MNHN Paris - Muséum National d'Histoire Naturelle (France); ALA - Atlas of Living Australia; SIO - Scripps Institution of Oceanography (USA); Invemar - Instituto de Investigaciones Marinas y Costeras (Colombia); ICMyL - Instituto de Ciencias del Mar y Limnología (Mexico); Smithsonian NMNH - National Museum of Natural History (USA); NHM London - Natural History Museum (UK).

Repository	All Records	Valid Records (VR)	VR (%)
OBIS	200,714	80,228	57.81
GBIF	210,266	43,475	31.33
O'Hara *et al.*	15,537	9,573	6.90
MNHN Paris	10,949	2,272	1.64
ALA	31,730	1,790	1.29
SIO	1,483	668	0.48
Invemar	3,347	535	0.39
ICMyL	3,193	211	0.15
Smithsonian NMNH	4,000	17	0.01
NHM London	2,520	0	0

Supplementary Table 2 | Pearson product-moment correlation coefficients between latitudinal patterns in observed ophiuroids species richness, number of absent species, number of sampling events and estimates of inventory completeness between OBIS dataset and OBIS + additional datasets.

	r	*p*-value
N° of observed species	0.93	< 0.001
N° of species absent	0.99	< 0.001
N° of sampling events	0.98	< 0.001
Sousa-Baena' estimate	0.53	0.002
Sample coverage estimate	0.89	< 0.001
SACs estimate	0.94	< 0.001

Supplementary Table 3 | Number of records of all taxonomic group after each step of the data cleaning process (columns). Valid coords: marine records with both coordinates different from zero or within the sea boundary; Species level: records with species-level identification; Valid name: records with scientific name accepted by World Register of Marine Species; Valid date: records with sampling date available or, if unavailable, from a unique sampling event; S: total species richness. Data retrieved on February 22-23, 2017 for Ophiuroidea and April 11-17, 2017 for other taxa.

Taxon	Initial records	Valid coords	Species level	Excluding fossils	Excluding duplicates	Valid name	Valid date	S
Ophiuroidea	483739	441008	320890	319863	150420	148006	138769	1793
Bivalvia	974505	964471	823313	823101	435392	434302	422318	3789
Gastropoda	947776	910292	656780	651386	436285	428644	416105	11104
Copepoda	3698045	3682786	1988043	1987995	879273	875608	874254	3983
Porifera	378990	376956	151759	151516	101761	101611	100704	4902
Rhodophyta	422822	419328	321915	314032	184938	184856	181741	2555
Amphipoda	605049	599342	392659	392555	244344	243634	235322	4327
Scleractinia	588274	583904	472722	472318	135679	134512	122812	1382
Elasmobranchii	1082665	1078155	917292	917292	543644	543637	540319	962
Scombridae	890339	888193	862855	862855	411446	411444	410358	52
Fraction eliminated		1.79%	26.51%	0.28%	33.81%	0.19%	0.93%	

Supplementary Table 4 | Impact of removing records with double zero coordinates on estimates of species with spatial gaps. Records: number of records with double zero coordinates (latitude and longitude exactly equal to zero); Species level: number of records with species-level identification; S: number of species; S-Gap: number of species with spatial gaps; Total gaps: number of species with spatial gaps in the final filtered dataset; Impact: percentage of species with 0-0 location records removed that have a spatial gap in the final filtered dataset.

Taxon	Records	Species level	S	S-Gaps	Min depth	Max depth	Total gaps	Impact (%)
Ophiuroidea	2	2	1	0	11.75	14.65	814	0.00
Bivalvia	21	21	4	3	-	-	1459	0.21
Gastropoda	5	4	3	0	0	11.75	3397	0.00
Copepoda	1	1	1	1	-	-	1319	0.08
Porifera	0	0	0	0	-	-	1083	0.00
Rhodophyta	104	71	55	28	-	-	861	3.25
Amphipoda	15	15	14	10	250	250	1194	0.84
Scleractinia	3	3	3	1	8.84	8.84	808	0.12
Elasmobranchii	41	37	25	12	0	50.5	463	2.59
Scombridae	1	1	1	0	0	0	34	0.00

Supplementary References

1. Herkt, K. M. B., Skidmore, A. K. & Fahr, J. Macroecological conclusions based on IUCN expert maps: A call for caution. *Glob. Ecol. Biogeogr.* **26,** 930–941 (2017).
2. Chao, A. & Jost, L. Coverage-based rarefaction and extrapolation: standardizing samples by completeness rather than size. *Ecology* **93,** 2533–2547 (2012).
3. Hsieh, T. C., Ma, K. H. & Chao, A. iNEXT: iNterpolation and EXTrapolation for species diversity. R package version 2.0.12. https://CRAN.R-project.org/package=iNEXT (2016).
4. Stropp, J. *et al.* Mapping ignorance: 300 years of collecting flowering plants in Africa. *Glob. Ecol. Biogeogr.* **25,** 1085–1096 (2016).
5. Sousa-Baena, M. S., Garcia, L. C. & Peterson, A. T. Completeness of digital accessible knowledge of the plants of Brazil and priorities for survey and inventory. *Divers. Distrib.* **20,** 369–381 (2014).
6. Yang, W., Ma, K. & Kreft, H. Geographical sampling bias in a large distributional database and its effects on species richness-environment models. *J. Biogeogr.* **40,** 1415–1426 (2013).
7. Tittensor, D. *et al.* Global patterns and predictors of marine biodiversity across taxa. *Nature* **466,** 1098–1101 (2010).
8. Oksanen, J. *et al.* vegan: Community Ecology Package. R package version 2.4-6. https://CRAN.R-project.org/package=vegan (2018).
9. Vandepitte, L. *et al.* Fishing for data and sorting the catch: Assessing the data quality, completeness and fitness for use of data in marine biogeographic databases. *Database* **2015,** 1–14 (2015).
